# How effective are interventions to improve social outcomes among offenders with personality disorder: a systematic review

**DOI:** 10.1186/s12888-017-1536-3

**Published:** 2017-11-17

**Authors:** Catriona Connell, Vivek Furtado, Elizabeth A. McKay, Swaran P. Singh

**Affiliations:** 1 Birmingham and Solihull Mental Health NHS Foundation Trust, Birmingham, UK; 2 University of Warwick, Coventry, UK; 3 Brunel University London, Uxbridge, UK; 4 Coventry and Warwickshire Partnership NHS Trust, Coventry, UK

**Keywords:** Personality disordered offenders, Social outcomes, Participation, Employment, Social functioning

## Abstract

**Background:**

Offenders with personality disorder are supported by health, criminal justice, social care and third sector services. These services are tasked with reducing risk, improving health and improving social outcomes. Research has been conducted into interventions that reduce risk or improve health. However, interventions to improve social outcomes are less clearly defined.

**Methods:**

To review the effectiveness of interventions to improve social outcomes we conducted a systematic review using Cochrane methodology, expanded to include non-randomised trials. Anticipated high heterogeneity of the studies informed narrative synthesis.

**Results:**

Eleven studies met inclusion criteria. Five contained extractable data. No high-quality studies were identified. Outcomes measured clustered around employment and social functioning. Interventions vary and their mechanisms for influencing social outcomes are poorly operationalised. Although change was observed in employment rates, there was no evidence for the effectiveness of these interventions.

**Conclusions:**

There is a lack of evidence for effective interventions that improve social outcomes. Further research is recommended to reach consensus on the outcomes of importance, identify the factors that influence these and design theoretically-informed and evidence-based interventions.

**Electronic supplementary material:**

The online version of this article (10.1186/s12888-017-1536-3) contains supplementary material, which is available to authorized users.

## Background

Personality disorder is highly prevalent among men and women with an offending history. In the United Kingdom, 64-78% of the adult male prison population and 50% of females meet diagnostic criteria for at least one personality disorder [1]. Personality disorder is the most common mental disorder in the probation population, affecting up to half of probationers [2]. Within high secure psychiatric facilities, personality disorder is definitely diagnosable in 57-77% of male patients [3]. People with an offending history and personality disorder (personality disordered offenders: PDOs) are a group whose difficulties come at a considerable cost to themselves, potential victims, the communities in which they live and return, and to society as whole who must meet the costs of service provision. PDOs experience worse physical and mental health, poorer quality of life, reoffend at higher rates and are overrepresented in the commission of serious further offences [4–6], indicating existing approaches may be overlooking important factors.

PDOs are supported by health, criminal justice, social care and third sector services. Whilst approaches have varied internationally and over time, a consistent theme is the requirement of services to reduce risk, improve health and improve social outcomes [7, 8].

Social outcomes are those that result from functioning effectively in society, for example participation through employment, family roles and independent living. In this paper, social outcomes are conceptualised as *participation*, as defined by the World Health Organization [9] as ‘involvement in a life situation’. Attention to participation among PDOs is vital for two reasons. Firstly participation in personally meaningful *and socially valued* (prosocial) activities is integral to functioning, health and social outcomes [9]. Secondly, in offender populations, participation is also associated with desistance and reduced risk of reoffending [10–12]. Conversely, offenders who do not participate in prosocial activities (e.g. remain unemployed or lack prosocial relationships) or participate in antisocial activities (gang affiliation, substance use) are at higher risk of reoffending [13].

Social outcomes for ex-offenders are poor. For example in the UK the employment rate is only 27% on leaving prison [14], and of those referred to support agencies only 16% found and kept employment for 6 months or more [15]. Research into interventions to facilitate participation and improve social outcomes amongst PDOs specifically is limited, despite this important contributor to health, quality of life and desistance often being mentioned as an aim of service providers.

### Objectives

The objective of the review was to determine the effectiveness of interventions to improve social outcomes among offenders with personality disorder.

## Methods

We conducted the review according to the stages outlined in the Cochrane Collaboration handbook for systematic reviews [16]. Review methods and inclusion criteria were pre-specified in a protocol and registered on PROSPERO: ID = CRD42016042304 [17].

### Eligibility criteria

We included English language studies reporting research where participants were adult offenders with personality disorder, reporting any intervention (e.g. psychological, pharmacological, occupational, social) and a social outcome, i.e. participation in a community setting. No limitations were placed on date or quality of research papers. Opinion pieces, commentaries or service descriptions, editorials, and publications addressing laws, policies and/or media reports were excluded.

Offender status was defined as having committed at least one criminal offence as reported from an official source or self-report. Personality disorder or psychopathy was considered present where participants had a formal diagnosis indicated by use of structured tool or justified method. Social outcome was defined as participating in any prosocial activity or engaging in a social role in a community setting (not prison/inpatient hospital) after encountering criminal justice services. For example, employment, volunteering, running a household, caring for children or being in an intimate relationship.

### Information sources

We searched databases for criminal justice, psychological, social, allied health and psychiatric research (Web of Science, SCOPUS, PubMed, EMBASE, AMED, CINAHL, ASSIA, PsycINFO, National Criminal Justice Reference Service (NCJRS) Abstracts Database, Cochrane collaboration, Campbell collaboration) and grey literature (theses, relevant reports, UK government documents). Original database searches were completed in July 2016. Automatic database searches were used to maintain an up-to-date review until September 2017. We reviewed reference lists of included studies and key papers. Relevant journals were surveyed on a regular basis.

### Search strategy

The search strategy was tailored to the requirements of each database with input from a search strategist to include terms pertaining to 1) personality disorder or psychopathy, and 2) offenders, and 3) participation in a community setting.

### Study selection

We imported database results into Endnote reference management software [18] and removed duplicates. All titles and abstracts were screened to determine if a citation met inclusion criteria by CC. A random selection of citations (*n* = 400, 22%) was independently reviewed by the second reviewer (VF). Inter-rater reliability reached substantial agreement, calculated using Cohen’s Kappa [19, 20]. Where agreement was not reached on inclusion by discussion the third reviewer (EAM) reviewed the material and gave a definitive judgement. Where insufficient information was available from the abstract the full text was obtained to determine if it met inclusion criteria.

### Data collection

A data extraction tool was refined after piloting to include: year of data collection, country of origin, the aim/hypothesis of the study, study design, inclusion criteria, participant demographics, personality disorder diagnosis method and prevalence within sample, offender status, participation outcome of interest, description of intervention, analysis method, and results. Due to the small number of included studies, CC extracted all the data which was checked by the second and third reviewers (VF, EAM).

### Risk of bias in individual studies

We appraised study quality using validated structured tools appropriate to study type [21–23]. Studies were not excluded based on quality due to the limited evidence in this area. This is considered in interpretation of the review findings.

### Summary measures

As social outcome and participation are rarely discussed in the literature, it was unlikely that these terms would be used as study outcomes. To identify relevant outcomes, a range of terms were anticipated, e.g. employment, education, intimate relationship, community roles and leisure activities. Terms were derived from the WHO International Classification of Functioning chapters on activity and participation [24].

### Data synthesis

The Cochrane Collaboration four-step method of narrative synthesis of effectiveness studies [25] was applied with consideration to the inclusion of non-RCT designs. The steps are as follows: 1) Develop a theory of how interventions operate, 2) Preliminary synthesis of findings, 3) Exploring relationships in the data within and between studies, 4) Assessing the robustness of the synthesis.

## Results

### Included studies

**Fig. 1:**
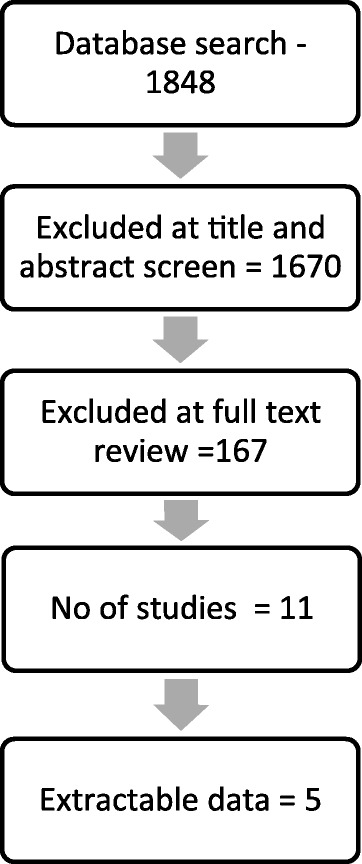
PRISMA Flow Diagram

The study selection is summarised in Fig. 1.

The search strategy provided a total of 1848 citations after adjusting for duplicates. After screening titles and abstracts, 178 were reviewed at full text. Of these, 11 met criteria for inclusion (see Table 1). Data could not be extracted from 6 studies because data relevant to PDOs could not be distinguished from the wider sample and the proportion of the sample with personality disorder or criminal history was below 60% [26–31].

**Table 1:** Studies meeting inclusion criteria

Author/s (Date published)	Title	Place of publication	Extractable data
Davidson et al. [33]	Cognitive behaviour therapy for violent men with antisocial personality disorder in the community: an exploratory randomized controlled trial	Psychological Medicine	Yes
Fones et al. [26]	The sexual struggles of 23 clergymen: A follow-up study	Journal of Sex and Marital Therapy	No Outcomes for PDOs not reported separately
Fortune et al. [35]	Clinical and economic outcomes from the UK pilot psychiatric services for personality-disordered offenders	International Review of Psychiatry	Yes
Grella et al. [27]	Follow-up of cocaine-dependent men and women with antisocial personality disorder	Journal of Substance Abuse Treatment	No Outcomes for PDOs not reported separately ASPD reported separately but criminal history unclear (probation supervision: 54% men, 43.7% women)
Krampen [37]	Psychotherapeutic processes and outcomes in outpatient treatment of antisocial behavior: An integrative psychotherapy approach	Journal of Psychotherapy Integration	Yes
Lindstedt et al. [31]	Mentally disordered offenders’ daily occupations after one year of forensic care	Scandinavian Journal of Occupational Therapy	No 27% PDOs Outcomes for PDOs not reported separately
Öhlin et al. [32]	Buprenorphine maintenance program with contracted work/education and low tolerance for non-prescribed drug use: a cohort study of outcome for women and men after seven years	BMC Psychiatry	Yes
Ryan et al. [28]	A follow up-study of probation service-approved premises residents in contact with mental health services	Journal of Forensic Psychiatry and Psychology	No PDOs 6.6% Outcomes for PDOs not reported separately
Simpson et al. [29]	Outcome of patients rehabilitated through a New Zealand Forensic Psychiatry Service: A 7.5 year retrospective study	Behavioral Sciences and the Law	No Outcomes for PDOs not reported separately
Whitehead et al. [38]	Time for a change: Applying the good lives model of rehabilitation to a high-risk violent offender	International Journal of Offender Therapy and Comparative Criminology	Yes
Wolff et al. [30]	Practice informs the next generation of behavioral health and criminal justice interventions	International Journal of Law and Psychiatry	No Outcomes for PDOs not reported separately

The five studies which contained extractable data are discussed for the remainder of the review. See Table 1 for included studies and those with extractable data. Four studies involving 94 participants reported social outcomes in the community for PDOs following an intervention. One study [32] did not specify the number of the 148 participants in their study who were PDOs.

### Study characteristics

Studies are presented in order of robustness of study design. The three cohort studies are presented in order of quality from high to low. The data extracted are summarised here. (See Additional file 1 for full data extraction).

One study used randomised controlled trial design (RCT) [33] to test the effectiveness of cognitive behavioural therapy (CBT) for reducing violence and aggression among 52 men with antisocial personality disorder (ASPD) in the community. Social functioning was included as an outcome measure, measured using the Social Functioning Questionnaire (SFQ) [34].

Öhlin et al. [32] report a prospective clinical study with an observational design involving 7-year follow up of 148 heroin dependent patients who received a voluntary multi-modal treatment including mandated employment. Social outcomes reported are rates of employment and subsidised education among those retained in treatment compared to those who dropped out. This is the only study to include women.

Fortune et al. [35] conducted a two-year prospective study of a cohort of 54 male service users from three forensic personality disorder services, 24 of whom were stated to be in the community. They collected baseline data in 2005-06 and followed up in 2007-08. These services were delivered by teams spanning medium secure units (MSUs) and the community, one of which explicitly stated an aim to assist patients to find participation opportunities in the local community. Similar to the other UK study [33], social functioning is taken as an outcome, though in this study it is measured using the Work and Social Adjustment Scale (WSAS) [36].

Krampen [37] conducted an observational cohort study to identify the five-year outcomes of long-term integrative psychotherapy for men referred for ‘acting out’ and ‘violence against intimates’. Psychotherapy was provided for an average 1 year (7-19 months) and included a range of clearly described techniques. Social outcomes were identified as change in employment rates, with employment defined as being ‘on the job’ (inferred to mean stable employment) for 2 years, and social adjustment although this is not defined or compared with a baseline score.

Finally, Whitehead et al. [38] present a case study, purposefully selected to illustrate the application of the Good Lives Model [39] in treatment for high risk offenders. Effectiveness was reported with qualitative details of both reducing risk and increasing prosocial participation via attending university, forming an intimate relationship and redefining social networks. The case study is a 28 year old Maori man (indigenous New Zealander) whose scores on the psychopathy checklist screening version (PCL-SV) [40] were reported to be indicative of high levels of psychopathy, and who had an extensive and serious offending history.

### Intervention descriptions

#### Cognitive behavioural therapy

Davidson et al. [33] tested cognitive behavioural therapy (CBT) developed for personality disorder [41] delivered in either 15 sessions over 6 months or 30 sessions over 12 months, each session lasting up to one hour. The authors outline that firstly, CBT encourages participants to engage in treatment through a cognitive formulation of their problems. Secondly, CBT focuses on beliefs, about self and others, and behaviours that impair social and adaptive functioning.

An element of CBT was included in two of the observational cohort studies [32, 37]. However, as part of a wider intervention the specific effect of CBT cannot be determined.

### Non-specified multi-modal treatment

Öhlin et al. [32] present a multi-modal intervention for heroin users. The treatment programme appeared to run indefinitely as all those not still within the programme at the 7 year follow up point were reported as ‘non-completers’. The treatment programme included five components. (1) Pharmacological treatment with buprenorphine to manage opioid addiction. (2) Prohibition of misuse of drugs. (3) Access to drug-free accommodation, although no further detail is given. (4) Achieving structured employment (work or studies). This element is not clearly described. The paper refers to working with a local employment agency, and that an existing ‘employment contract’ was required for inclusion in the programme. (5) Psychosocial treatment sessions to modify drug use and ‘prevent passivity’ which included manual-based cognitive-behavioural therapy, psychodynamic or family-oriented counselling.

### Multi-disciplinary forensic psychiatric services

Fortune et al. [35] evaluated outcomes for patients treated in three MSUs and associated community services. The authors state the service aims to provide treatments to reduce the risk of re-offending, address mental health needs and improve social functioning. One of the community services was a residential service provided by a local housing organisation that provided social care for eight residents. This included assistance in exploring local opportunities for education, employment and other activities. What was done on an inpatient basis in preparation or by the other community teams to target social functioning is unclear.

### Integrative psychotherapy

Krampen [37] describes integrative psychotherapy as including cognitive-behavioural, relaxation and psychodynamic methods. Treatment principles, the four therapeutic aims and the techniques of the psychotherapy delivered are described in depth. These included (1) Enhanced social-emotional skills, empathy and morality, (2) Reduced psychophysiological arousal in favour of impulse control and mastery, (3) Developing adaptive self-statements, and (4) Reconstructing attachment abilities, trust, and social relationships. (See Additional file 1 for further details).

### Good lives informed psychological intervention

Whitehead et al. [38] describe treatment informed by the Good Lives Model which aims to provide the internal and external conditions that make successfully achieving a good life possible. Five phases of treatment are described with reference to case material as follows: 1) Identifying life goals and the motivation for pursuing them; 2) Defining desired identity and determining the barriers/opportunities to achieving this; 3) Producing a good lives informed formulation; 4) Developing a plan to equip the offender with values, attitudes, skills and resources to achieve their goals in a prosocial way; 5) Enacting the plan, including undertaking any interventions to address criminogenic barriers such as substance use or attitudes towards violence. Components relevant to achieving social outcomes are not made explicit, though there appears to be elements of practical assistance, counselling and guidance in addition to what is covered in the therapy sessions.

### Results of individual studies

Davidson et al. [33] use intention to treat principles in their analysis. SFQ scores were taken at baseline and the participants last attended session. Mean difference in score on the SFQ were calculated, adjusted for baseline levels. There was no significant difference in social functioning between the combined CBT groups (those who received either 6 or 12 months) and treatment as usual (TAU) group. Mean difference was −0.7 (95% CI = −3.3 to 1.8), *p* = 0.54. The authors report a trend toward significance for those who received 6 months of CBT to have improved social functioning compared to TAU (*p* = 0.08, data not shown). However, they also acknowledge that the study is underpowered to reliably detect change.

Öhlin et al. [32] report frequency counts and percentages of those in employment at the start and end of the 7-year period, and compare results for those retained in treatment compared to those who dropped out. They offer no statistical analysis on this outcome. Reasons for drop-out are not given. They report that 69% of patients were employed in a regular job at 7 years compared to 22% at baseline and 29% earned their living by a subsidised wage compensation compared to 9.5% at baseline. 2% conducted academic studies. Proportionally more women than men were in work or education (70% vs 60%) but there was a 30% improvement for both sexes in movement from precarious work to employment in the regular labour market. Subsidised wage compensation increased by 19% during the first 2 years of follow-up. They suggest all participants who dropped out lost employment soon after and did not resume, compared to those who sustained their engagement with treatment and retained employment. Whilst showing positive trends, as work was a mandated component of the intervention it is not possible to ascertain if change would be sustained on completion. As there was no control group change cannot be attributed to the intervention.

In assessing social function, Fortune et al. [35] used a paired t-test to detect statistically significant change on WSAS scores at baseline and 24 months. For the group reported to be in the community, initially 24 men, there was no significant difference in social functioning at 6 or 24 months. Mean WSAS at baseline =20.42 SD (12.12). Mean at 6 months 19.53 (SD 10.97), *T* = 0.81, *p* = 0.43. Mean at 24 months = 14.5(8.3), *T* = 1.04, *p* = 0.33.

Like Öhlin et al. [32], Krampen [37] reports pre-and post-employment rates in frequency counts but no further statistical analysis. For the ASPD subgroup, those who had been in stable employment for 2 years increased from 41% (*n* = 7) to 71% (*n* = 12). At follow-up, 76% (*n* = 13) had what the author refers to as social adjustment, although offers no pre-test score or explanation of what this is based upon. As there was no control group change cannot be attributed to the intervention.

Whitehead et al. [38] use no formal analysis procedures, reporting a case study and formulation to make inferences about treatment effectiveness and the potentially active mechanisms. The authors frame intervention as a success, particularly in comparing violent reoffending with that occurring during the participant’s last parole. The participant commenced university and a diving qualification, but did not complete either. He had started learning to drive but completion was not reported. The participant was also reported to be in an intimate relationship and to have had success in establishing a new prosocial peer group. As single case study, it is not possible to ascribe change to the intervention.

### Risk of bias / quality appraisal of individual studies

Studies were appraised using the Downs and Black tool [22] with the exception of Davidson et al. [33] which was the only RCT, and was thus also assessed for bias using the Cochrane tool [23].

Davidson et al. [33] was a small-scale feasibility study in which only the data collectors were blind to the intervention groups. The inability to conceal psychotherapy interventions from participants and practitioners is well documented. The small sample size (total *n* = 52) mean there was insufficient power in statistical analysis. The risk of bias overall was rated as medium.

Öhlin et al. [32] provide the most comprehensive report of the observational cohort studies. However, details of the intervention itself are limited. There was no control group, it is unclear how long treatment lasted and there was no reported adjustment for length of follow up. The study is considered high risk of bias.

Fortune et al. [35] was the only cohort study to use statistical tests to determine the significance of any change in the outcome of interest (social functioning). However, the description of the intervention is lacking, and was delivered by three different real world teams. Refusal to participate was high (39%) limiting confidence in the representativeness of their sample and there was no control group. Risk of bias is high.

Krampen [37] was judged to be very high risk of bias because of the limited reporting of key criteria to judge the study. For example, confounding factors, description of when measures were taken and by whom, and whether those lost to follow up had different characteristics. There is no control group.

Whitehead et al. [38] reports a purposively selected case, deliberately chosen to illustrate the that intervention informed by the Good Lives Model can be effective with challenging PDOs. High risk of bias is evident in the stated aim of the authors to make this point.

### Synthesis of results

Due to limitations in the designs of the studies and high heterogeneity, meta-analysis was not possible. A narrative synthesis identified the outcomes measured, the types of interventions, the mechanisms by which interventions were hypothesised to improve social outcomes, and their effectiveness in achieving that aim. See Table 2.

**Table 2:** Result synthesis

Study	Social outcome	Intervention	How intervention may impact participation and social outcome	Effectiveness
Davidson et al. [33]	Social functioning measured with SFQ	CBT for personality disorder	Therapy focuses on beliefs about self and others, and behaviours that impair social and adaptive functioning Attitude and behaviour that blocks successful participation ‘challenged’ and reduced, which may result in improved social functioning.	No significant difference
Fortune et al. [35]	Social functioning measured using WSAS	MSU and community treatment in 3 teams. One service helped explore local opportunities for participation (education, employment and other activities).	Unclear Practical assistance/support to overcome barriers to accessing real world experiences of participation. Real world experiences allow for developing skills and abilities in response to challenges in live settings that can be continued in future participation.	No significant difference
Krampen [37]	Employment defined as being ‘on the job’ for at least two years	Long-term integrative psychotherapy Including: Resource activating interventions, mastery-oriented interventions and consciousness-creating interventions	Not explicit which interventions (see additional file 1 for full detail) or treatment objectives relate to employment specifically. Overall therapy objectives included enhanced social emotional skills, empathy and morality; increased impulse control and mastery; producing adaptive self-statements; reconstructed attachment ability, trust and social relationships and developing prosocial peer networks. The above may build capacities to better cope with the social and emotional challenges of a work environment, and solve problems by modelling behaviour from prosocial networks.	Increased employment rate. Difference can’t be attributed to intervention
Öhlin et al. [32]	Employment Either in competitive employment or ‘subsidised wage compensation’	Multi-modal treatment including employment advisors ‘Support radical lifestyle change’	Unclear how the intervention got participants into a job, and what role was played in sustaining this during and post intervention. If participants were provided with practical assistance to gain and sustain employment this may involve embedding a new routine, experiencing work and learning adaptive skills to sustain this role.	Increased employment rate. Difference can’t be attributed to intervention
Whitehead et al. [38]	Mixed University, prosocial leisure and relationship	Psychologist and other team members (e.g. Maori mentor) using Good Lives Model	Motivation to engage and sustain change in participation is enhanced by producing cognitive dissonance between desired identity and current situation. Interventions orientated around imparting values, attitudes, skills resources needed to make most of opportunities and overcome barriers Staff practical support, information giving (e.g. finding course information) and orchestrating positive life events may enable the offender to initiate participation and then develop competences/identity to continue independently and generalise to other activities	Began participating Difference can’t be attributed to intervention

### Intervention outcome and effectiveness

#### Social functioning

Three studies attended to social functioning. Davidson et al. [33] measured changes on the Social Functioning Questionnaire [34] following CBT. No significant difference was found after CBT although the study was underpowered. Fortune et al. [35] similarly found no significant change in scores on the Work and Social Adjustment Scale [36] during 2 years of multidisciplinary forensic mental health intervention. Whitehead et al. [38] demonstrated the results of intervention informed by GLM in a single case. The participant developed prosocial networks, leisure pursuits and an intimate relationship. As a single case, change cannot be attribute to GLM informed treatment.

There is no evidence that the reported interventions increased social functioning.

### Employment and education

Three studies report on employment and education. Krampen [37] identified presence or absence of a 2-year period of job stability. Öhlin et al. [32] referred to employment as including competitive work and receiving subsidised wage compensation, without specifying length of employment. They also report those who went on to education. Both studies showed an increase in employment rates. However as observational studies, it is not possible to attribute change to the interventions. Whitehead et al. [38] reported participation in education as an outcome though with a GLM approach, the outcome of interest will always be individually defined. Successfully commencing university is cited as a success though limitations of the case study design are acknowledged.

There is evidence that employment can be achieved by PDOs over time. However, the study designs prevent attribution of change to the interventions.

### Intervention mechanisms

Three potential mechanisms that supported PDOs to participate in prosocial activities were identified; skill development, defining a prosocial goal and identity, and real world experiences achieved through practical assistance.

Two interventions explicitly mentioned developing skills relevant to participation in prosocial activities. The integrative psychotherapy intervention described by Krampen [37] included social and emotional skills training and anger and self-control training, whilst Whitehead et al. [38] refer to equipping their participant with values, attitudes, skills and resources that supported success in achieving his goals through prosocial means. Based on the assumption that skill deficits were a barrier to success, skill development may give PDOs the ability to overcome barriers to accessing prosocial activities; better cope with challenges that disrupt participation; or develop new strengths that support sustained participation.

Two studies described prosocial goals and identity. Krampen [37] refer to developing ‘life projects’ in interventions that are ‘consciousness creating’. Whitehead et al. [38] describe how they drew out prosocial goals and orientated treatment around achieving these. Enabling a PDO to identify with prosocial roles and working towards achieving this aim may operate to achieve change by enhancing motivation and engagement for change, and by identifying and addressing relevant barriers and opportunities for participation.

Three studies referred to using real-word participation achieved through practical assistance. Öhlin et al. [32] explicitly included employment in their intervention, although how this was delivered and whether this was integral to treatment outcome is not well described. In Fortune et al. [35], one of the three community services provided practical assistance to access local opportunities for participation. Whitehead et al. [38] provided practical assistance to attain participation experiences in university. Through supported participation, service users may have learned skills, begun to view themselves differently and experienced enhanced motivation to pursue their prosocial goals through mastery experiences. However, practical assistance may not equip the PDO to continue participation independently, given the unemployment rates among the dropouts in the study by Öhlin et al. [32], and the lack of statistically significant change in social functioning by Fortune et al. [35]. There is potentially a need for PDOs to learn to generate their own participation if positive social outcomes are to be sustained.

## Discussion

This review evaluated the effectiveness of interventions to improve social outcomes among PDOs. It conceptualised social outcomes as analogous to the World Health Organisation concept of participation [9]. Five studies were included in the review. Narrative synthesis was conducted due to high heterogeneity. Reported outcomes could be grouped into employment and social functioning. There were three potential mechanisms identified in the interventions that may improve participation. There was no evidence for significant change in social functioning and although rates of employment were noted to increase, the quality and designs of the studies prevent attribution to the interventions.

To maximise effectiveness, interventions should be developed based on evidence of what the relevant influencing factors are, and a theory of the mechanisms by which they operate to bring about a desired outcome [42]. However, interventions identified in this review lacked theoretical explanations of how they may work to improve participation and there was variation in the social outcomes reported.

Attention to employment as a relevant outcome is consistent with the literature that identifies employment as an important factor in desistance from crime in offender populations [43, 44] and in protecting against serious reoffending among mentally disordered offenders [10]. Within the health literature, employment is identified as an indicator and facilitator of mental health and wellbeing [45]. However, employment only reflects a small component of participation, which includes many activities essential for survival, health and social inclusion such as leisure, domestic responsibilities and civic involvement. Social functioning appears more closely related to participation defined in this way. Consensus around measures of participation in mental health is lacking, complicated by ongoing debates on operationalising participation that have resulted in multiple measures being developed [46]. Until a stronger consensus is achieved on the outcome of interest and its measurement, there is a risk of continued heterogeneity in intervention research that prevents synthesis of trial results.

Three potential mechanisms of change were identified from synthesis of the study descriptions. The first of these was skill development, which may be based on the hypothesis that lack of social, emotional or practical skills impede participation. Skills training is well established in criminal justice programming, including specific programmes for PDOs (e.g. [47]). However, interventions are institutionally based and research is required to determine if any skills learned are transferred and applied to participation in the community, or ‘real-world’, on release. The second mechanism was facilitating change in values and identity through supported prosocial goal attainment and validation of efforts. This approach is increasingly adopted in forensic practice to address motivation and engagement for risk-focused intervention, by framing offending as a barrier to achieving prosocial goals [48]. ‘Volitional realignment’ towards prosocial goals and identity change are argued to occur through mastery of new prosocial activities by those practicing from an occupational perspective [49]. Interventions using this approach are yet to be proven effective. The final mechanism was practical assistance given to compensate for participants’ difficulties, for example taking someone to a leisure centre. Whilst this has an immediate effect, it does not impart a change in the individual him or herself, and thus may not support continued participation on a long-term basis. This approach is consistent with the Individual Placement and Support model, which has been shown to be effective in supporting individuals with serious mental illness into employment [50]. However, whether employment is then sustained is less clear from the literature. Similarly, in employment interventions for ex-offenders, only 16% retained any employment achieved for longer than 6 months [15]. This is an important consideration in providing interventions of long-term effectiveness and when working with individuals with personality disorder, whose difficulties may never ‘resolve’, as can be the case for people with psychotic disorders.

The heterogeneity of the studies in this review indicate that a theoretically-informed exploration of the factors influencing participation is required, before systematic development and evaluation of interventions that are likely to be effective can be conducted [51]. Identifying the influencing factors can be approached from multiple perspectives. For example, identifying the features of personality disorder, such as traits or severity, that influence social outcomes and thus targeting treatment at the modifiable traits or symptoms. An alternative approach would be to identify which components of participation influence social outcomes among PDOs. Intervention would then be focused on modifying these components of participation, rather than attempting to ameliorate signs and symptoms of disorder/s. This approach is more familiar to rehabilitation professionals, who advocate the WHO position that health and functioning are achievable irrespective of disability, disorder or disease [9].

The WHO International Classification of Functioning (ICF [9]) provides an internationally recognised framework for describing and classifying strengths and difficulties in participation in great detail. However, it does not explain how different factors interact to produce participation, and as discussed, operationalisation of participation remains contested. The Theory of Human Occupation and its related conceptual practice model [52] explains of how participation is achieved, experienced, maintained and changed, and has valid and reliable measures for associated and influencing factors. Although it has not been tested specifically with PDOs, the Theory of Human Occupation, like the ICF, is based on universal principles and its utility is evident in its use in international forensic research and practice [31, 49, 53]. This may present a starting point for identifying relevant factors and the mechanisms of change that can be facilitated through intervention.

### Limitations

The review was conducted according to a pre-specified protocol informed by Cochrane guidelines for conducting systematic reviews [16]. In a clarification to the protocol, studies were included where results were not differentiated for PDOs only where at least 60% of the sample had personality disorder/psychopathy and at least 60% had committed an offence. Previous systematic reviews have set a level of 70% when taking outcomes from a mixed sample [54]. Because of the known high prevalence of undiagnosed personality disorder among offenders, a slightly lower percentage was considered acceptable. These criteria permitted inclusion of the study by Öhlin et al. [32].

The feasibility of RCTs is limited in testing complex interventions involving prolonged therapy, such as psychotherapy or occupational therapy. This informed the decision not to exclude studies on the grounds of quality. Due to the inclusion of low quality studies, conclusions drawn from this review must be interpreted cautiously.

## Conclusions

No interventions identified were designed to specifically improve social outcomes in the community among PDOs. There is some evidence that employment can be achieved although changes cannot be attributed to interventions due to the study designs used. There was no evidence for interventions aiming to improve social functioning.

There is a sizeable gap in the literature reporting interventions to improve social outcomes, describing the mechanisms by which they are proposed to work, and testing effectiveness. This is further constrained by the focus on different outcomes and variation in how they are measured. Consequently, services for PDOs are unable to apply evidence-based interventions that are likely to increase social outcomes among offenders with personality disorder in the community.

### Implications for practice

Services and practitioners working with PDOs in the community currently lack evidence on which to base interventions that specifically target social outcomes. Service providers may consider interventions that are orientated towards achievement of a personally meaningful prosocial identity; target skill deficits that impact on successfully maintaining participation in employment and social relationships; or provide practical assistance to access prosocial roles that have previously been unfamiliar.

### Implications for research

Evidence for effective interventions to improve social outcomes is lacking. Further research is required to identify the factors that influence participation, develop interventions to target these, and to test their effectiveness.

## Additional file

Additional file 1: Data extraction. Table of all the data extracted from included studies. (XLSX 42 kb)

## Abbreviations

ASPD: Antisocial personality disorder
CBT: Cognitive behavioural therapy
GLM: Good Lives Model
MSU: Medium secure unit
PCL-SV: Psychopathy checklist - screening version
PDO: Personality disordered offender
RCT: Randomised controlled trial
SFQ: Social functioning questionnaire
TAU: Treatment as usual
WSAS: Work and social adjustment scale
